# Therapeutic validation of an orphan G protein‐coupled receptor: The case of GPR84

**DOI:** 10.1111/bph.15248

**Published:** 2020-09-17

**Authors:** Sara Marsango, Natasja Barki, Laura Jenkins, Andrew B. Tobin, Graeme Milligan

**Affiliations:** ^1^ Centre for Translational Pharmacology, Institute of Molecular, Cell and Systems Biology, College of Medical, Veterinary and Life Sciences University of Glasgow Glasgow UK

**Keywords:** allosteric ligand, GPCR, GPR84, inflammation, orphan receptor, orthosteric ligand

## Abstract

Despite the importance of members of the GPCR superfamily as targets of a broad range of effective medicines many GPCRs remain poorly characterised. GPR84 is an example. Expression of GPR84 is strongly up regulated in immune cells in a range of pro‐inflammatory settings and clinical trials to treat idiopathic pulmonary fibrosis are currently ongoing using ligands with differing levels of selectivity and affinity as GPR84 antagonists. Although blockade of GPR84 may potentially prove effective also in diseases associated with inflammation of the lower gut there is emerging interest in defining if agonists of GPR84 might find utility in conditions in which regulation of metabolism or energy sensing is compromised. Here, we consider the physiological and pathological expression profile of GPR84 and, in the absence of direct structural information, recent developments and use of GPR84 pharmacological tool compounds to study its broader role and biology.

**LINKED ARTICLES:**

This article is part of a themed issue on Structure Guided Pharmacology of Membrane Proteins (BJP 75th Anniversary). To view the other articles in this section visit http://onlinelibrary.wiley.com/doi/10.1111/bph.v179.14/issuetoc

**Abbreviations**
2‐HTP: 2‐(hexylthio)pyrimidine‐4,6‐diol
6‐OAU: 6‐n‐octylaminouracil
APP‐PS1: a murine model of Alzheimer's disease
DIM: 3,3′‐diindolylmethane
GLPG1205: 9‐cyclopropylethynyl‐2‐((S)‐1‐[1,4]dioxan‐2‐ylmethoxy)‐6,7‐dihydropyrimido[6,1‐a]isoquinolin‐4‐one
IBD: inflammatory bowel disease
ICL3: intracellular loop 3
MCFAs: medium‐chain fatty acids
NAFLD: non‐alcoholic fatty liver disease
PAM: positive allosteric modulator
PMA: phorbol myristate acetate
PMN: polymorphonuclear leukocyte
PNL: partial sciatic nerve ligation

## 1 INTRODUCTION

GPR84 is a poorly characterised G_i_‐coupled, class A GPCR mainly expressed by immune cells, including monocytes, macrophages and neutrophils in the periphery, and microglia in the brain (Wojciechowicz & Ma'ayan, 2020). Because GPR84 transcript is up‐regulated in many pro‐inflammatory conditions, potential therapeutic opportunity in targeting this receptor in inflammatory conditions, including ulcerative colitis and fibrotic diseases, has been suggested (Gagnon et al., 2018; Suzuki et al., 2013; Vermeire et al., 2017; Wojciechowicz & Ma'ayan, 2020). Identified almost two decades ago (Wittenberger, Schaller, & Hellebrand, 2001; Yousefi, Cooper, Potter, Mueck, & Jarai, 2001), human GPR84 encodes a protein of 396 amino acids that shares 85% identity with the protein encoded by murine Gpr84. The extensive intracellular loop 3 (ICL3) provides most of the differences between these orthologues, while the predicted transmembrane domains have only 14 amino acid differences. Although it is widely accepted that medium‐chain fatty acids (MCFAs), particularly those with 10–12 carbon acids (decanoic acid [C10], undecanoic acid [C11], and lauric acid [C12]), can bind to and activate GPR84 (Nikaido, Koyama, Yoshikawa, Furuya, & Takeda, 2015; Southern et al., 2013; Suzuki et al., 2013; Wang, Wu, Simonavicius, Tian, & Ling, 2006), officially, GPR84 remains an orphan receptor (Sharman et al., 2011). This reflects that MCFAs display only modest potency in activating GPR84. In recombinant expression systems, the reported potency of MCFAs to either inhibit forskolin‐stimulated cAMP production or to stimulate [^35^S]guanosine 5‐*O*‐(3‐thiotriphosphate) ([^35^S]GTPγS) binding via GPR84 is in the low micromolar range. However, millimolar concentrations of C10–C12 fatty acids are required to induce secretion of the pro‐inflammatory cytokine IL‐12 p40, by LPS (which acts to up‐regulate levels of both mRNA and GPR84 protein; Mancini et al., 2019) in RAW264.7 cells. This suggests, as is often the case with various agonist studies, that estimates of agonist potency in transfected cell systems (Table 1) incorporate various levels of receptor reserve and often markedly overestimate ligand binding affinity at the receptor (Bolognini et al., 2019; Bradley et al., 2018). Because of this, we will avoid ascribing absolute agonist affinity values unless these have been generated by a reliable method, such as mathematical analysis of operational models (Ehlert, 2005) of the extent of allosteric modulation between ligands (Al Mahmud et al., 2017) that bind to distinct sites of GPR84 (Table 1) and limit comments to relative potency of compounds where these have been assessed in parallel using the same experimental system, for example, direct measures of EC_50_ employing functional assays of second messenger regulation or G‐protein activation in cell lines transfected to express an orthologue of GPR84. As such, levels of MCFAs circulating in the plasma (approx. 0.5 mM) might be too low to substantially activate GPR84 in vivo (Al Mahmud et al., 2017; Mancini et al., 2019).

**TABLE 1:** Agonist ligands with activity at GPR84

Compound name	Reported potency (EC_50_)	Model system (in vitro, ex vivo)	Comment	References
**Orthosteric**				
Decanoic acid (C10)	**Human:** 0.8–20.0 μM^b^ 4.5–25.1 μM^a^ 48 mM^a^	‐Recombinant system ‐RAW264.7 cells ‐Mouse bone marrow‐derived macrophages ‐Mouse primary cultured microglia ‐3T3‐L1 adipocytes		Wang et al., 2006 Nagasaki et al., 2012 Suzuki et al., 2013 Southern et al., 2013 Nikaido et al., 2015 Pillaiyar et al., 2017 Wei, Tokizane, Konishi, Yu, & Kiyama, 2017 Al Mahmud et al., 2017 Recio et al., 2018 Puengel et al., 2020 Lucy et al., 2019
Embelin	**Human:** 89–200 nM^b^ 0.63 μM^a^ 0.4 μM^c^ **Mouse:** 220 nM^b^	‐Recombinant system ‐Human monocyte‐derived macrophages ‐Mouse peritoneal macrophages ‐Human blood‐derived neutrophils ‐Mouse blood‐derived neutrophils ‐Mouse primary cultured microglia ‐Human monocyte ‐Rat neutrophils		Hakak, Unett, Gatlin, & Liaw, 2007 Southern et al., 2013 Al Mahmud et al., 2017 Wei et al., 2017 Pillaiyar et al., 2017 Gaidarov et al., 2018 Puengel et al., 2020
6‐OAU	**Human:** 14–438 nM^b^ 1.74–11 μM^c^ 512 nM^a^	‐Recombinant system ‐Mouse primary cultured microglia ‐Bone marrow‐derived macrophages ‐Human peripheral polymorphonuclear leukocyte ‐U937 cells differentiated into macrophage‐like cells ‐Human monocytes		Suzuki et al., 2013 Liu et al., 2016 Zhang, Yang, Li, & Xie, 2016 Wei et al., 2017 Recio et al., 2018 Lucy et al., 2019
PSB‐1584	**Human:** 5 nM^b^ 3.2 nM^c^	‐Recombinant system	Tritium‐labelled form of this compound is available ([^3^H]PSB‐1584)^d^	Pillaiyar et al., 2018 Köse et al., 2020
2‐HTP (Compound 1 or ZQ‐16)	**Human:** 1–144 nM^b^ 0.79–28.8 nM^a^ 597 nM^c^ **Mouse:** 30.2 nM^a^	‐Recombinant system ‐THP‐1 monocytes ‐RAW264.7 cells ‐Mouse bone marrow‐derived neutrophils ‐Human blood‐derived neutrophils ‐Human monocytes ‐Human monocyte‐derived macrophages ‐Mouse bone marrow‐derived macrophages		Zhang et al., 2016 Liu et al., 2016 Al Mahmud et al., 2017 Sundqvist et al., 2018 Mancini et al., 2019 Lucy et al., 2019
DL‐175	**Human:** 33 nM^b^	‐Recombinant system ‐Mouse bone marrow‐derived macrophages ‐U937 cells differentiated into macrophage‐like cells ‐Human monocytes	Displays bias agonism for G_i_‐protein signalling pathway over arrestin recruitment compared with 6‐OAU	Lucy et al., 2019
**Allosteric**				
DIM	**Human:** 0.25–1.25 μM^b^ 0.5–1 μM^a^ 5.9 mM^a^ **Mouse:** 4.26 μM^a^	‐Recombinant system ‐RAW264.7 cells	PSB‐15160 and PSB‐16671 are DIM analogues that display bias compared with DIM towards G_i_‐mediated adenylyl cyclase inhibition over arrestin recruitment^e^	Wang et al., 2006 Nikaido et al., 2015 Zhang et al., 2016 Al Mahmud et al., 2017 Pillaiyar et al., 2017 Mancini et al., 2019

*Note:* Note comments in the text about reported potency values and potential issues of receptor reserve.

^a^ Potency values were generated using [^35^S]GTPγS assay.

^b^ Potency values were generated using cAMP assay.

^c^ Potency values were generated using β‐arrestin recruitment assay.

^d^ Köse et al. (2020).

^e^ Pillaiyar et al. (2017) and Mancini et al. (2019).

Here, as well as discussing the expression profile of GPR84 in physiological and pathophysiological conditions, we will consider the portfolio of available ligands that can be used as tool compounds to study the function and biology of GPR84 and how limitations of these are currently restricting a full understanding of the therapeutic potential of this receptor. As noted above, although MCFAs certainly can activate GPR84, the modest potency of these in native systems has promoted efforts to identify and characterise other, more potent agonists. Moreover, as GPR84 is linked to various inflammatory diseases, efforts have also been made to identify antagonists to assess if these might have therapeutic utility in such settings.

**FIGURE 1:**
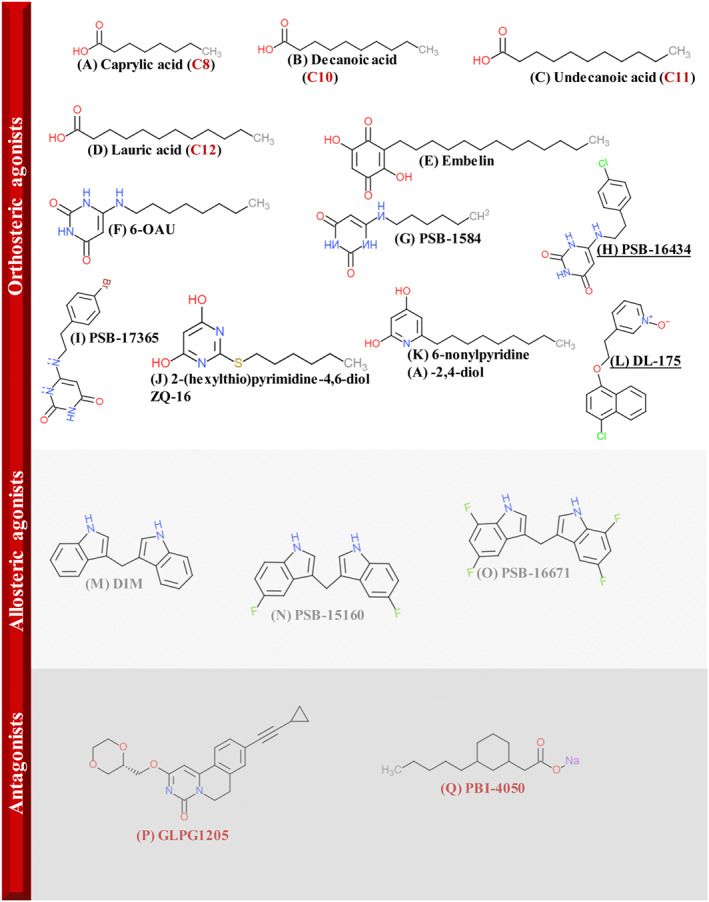
Chemical structures of low MW GPR84 ligands. The chemical structure of some widely used ligands with actions at GPR84 is shown. The alphabetical labelling system (A–O) is used to define which ligands were employed in studies highlighted in Figure 2 and Tables 1 and 2

It is assumed here that MCFAs act as orthosteric agonists, although while GPR84 remains classified as an orphan GPCR, this must remain a presumptive definition (Figure 1 and Table 1). Of key importance is that, although MCFAs do activate GPR84, equivalent fatty acid amides do not (Nikaido et al., 2015), and the requirement for the acid function was also observed when decanoic acid (C10) was replaced with its ester, methyl decanoate (Al Mahmud et al., 2017). Although this defines the importance of the acid function for binding and/or activation, in the absence of suitable atomic‐level structures, the potential orientation of the MCFA in the binding site remains a matter of conjecture. In class A GPCRs, the orthosteric binding site is typically a deep pocket leading from the extracellular side of the receptor with which endogenously produced ligands interact (Congreve, de Graaf, Swain, & Tate, 2020). By contrast, binding sites that are topographically distinct from the orthosteric site are generically described as allosteric (Congreve et al., 2020). Given that other class A GPCRs that respond to either short‐chain (free fatty acid receptors 2 and 3) or long‐chain (free fatty acid receptors 1 and 4) fatty acids have specific arginine residues as part of the orthosteric binding pocket that act to co‐ordinate the carboxylate of the appropriate fatty acid (Milligan, Shimpukade, Ulven, & Hudson, 2017), Al Mahmud et al. (2017) assessed whether this might also be true for GPR84, even though this receptor is not closely related to any of the currently IUPHAR‐accepted free fatty acid receptors (Alexander, Christopoulos et al., 2019). A chimeric homology model that noted and took into account the high similarity of the second extracellular loop of GPR84 with that of rhodopsin, for which atomic‐level structures were known, suggested that Arg^172^ from this region would potentially face into the binding pocket and anchor the fatty acid carboxylate (Tikhonova, 2017). Mutation of this residue to alanine, and indeed even to lysine, abolished function of MCFAs. By contrast, the potency of a known allosteric agonist (see later) of GPR84, 3,3′‐diindolylmethane (DIM), was unaffected by these mutations (Al Mahmud et al., 2017). Co‐ordination of the fatty acid carboxylate by this arginine implied that the alkyl chain of the fatty acid would penetrate down into the orthosteric cavity of the receptor with the carboxylate at the extracellular interface. In contrast, earlier mutagenesis and homology modelling studies by Nikaido et al. (2015) concluded that the opposite orientation of the fatty acid was more likely, with the carboxylate reaching downwards into the receptor. Mutagenesis and homology modelling studies by Köse et al. (2020) have supported this orientation, showing the fatty acid carboxylate in the binding cleft and forming hydrogen‐bond interactions with Tyr^69^, Asn^104^, and Asn^357^. In this model, Arg^172^ contributes indirectly to agonist–receptor interaction, possibly by playing a role in initial ligand recognition (Köse et al., 2020). It also appears that MCFAs with a hydroxyl group at the 2‐ or 3‐position can activate GPR84 more potently than non‐hydroxylated MCFAs (Suzuki et al., 2013). Hydroxylation at other positions on the fatty acid alkyl chain is also consistent with activation of the receptor (Kaspersen, Jenkins, Dunlop, Milligan, & Ulven, 2017).

## 2 ORTHOSTERIC AGONISTS

### 2.1 Embelin (2,5‐dihydroxy‐3‐undecyl‐1,4‐benzoquinone)

Embelin (2,5‐dihydroxy‐3‐undecyl‐1,4‐benzoquinone) is a natural product derived from the plant *Embelia ribes*, used in traditional Chinese medicine for treatment of diverse conditions, such as gastrointestinal and inflammatory diseases (Gaidarov et al., 2018). It was first described in the patent literature as a potent and efficacious GPR84 agonist using inhibition of cAMP assays (Hakak et al., 2007). Although appreciated for a period of time to have such activity by those working in the field, it was a number of years before this was confirmed in the peer‐reviewed literature (Al Mahmud et al., 2017; Southern et al., 2013; Wei et al., 2017). Embelin consists of a polar dihydroxybenzoquinone head group and an 11‐carbon alkyl chain tail (Figure 1). Truncation of the alkyl chain length to 7 or 8 carbons results in increased potency, whilst agonist activity is lost in derivatives containing very short (C3) or very long (C15) alkyl chains (Gaidarov et al., 2018). Embelin has a range of biological activities including inhibition of the X‐linked inhibitor of apoptosis protein and activation of caspase 9, as well as antioxidant properties (Nikolovska‐Coleska et al., 2004). Even at other GPCRs, embelin has been noted to have as potent actions, as at GPR84, but rather as a blocker; for example, at the chemokine receptor CXCR2 (*K*_*i*_ = 93 nM) and the adenosine A_3_ receptor (*K*_*i*_ = 1.4 μM). However, at least at these receptors, the C7 derivative reportedly lacks activity (Gaidarov et al., 2018). As such, embelin and derivatives have been used to characterise roles of GPR84 in a variety of settings (Table 1) (Al Mahmud et al., 2017; Gaidarov et al., 2018; Wei et al., 2017).

### 2.2 6‐OAU (6‐(octylamino) pyrimidine‐2,4(1*H*,3*H*)‐dione)

6‐OAU (6‐(octylamino) pyrimidine‐2,4(1*H*,3*H*)‐dione) is a further example of a GPR84 agonist characterised by a polar head group and alkyl tail (Figure 1 and Table 1) (Recio et al., 2018; Suzuki et al., 2013; Wei et al., 2017). 6‐OAU is able to induce chemotaxis of human polymorphonuclear leukocytes (PMNs) and macrophages and to promote production of the pro‐inflammatory cytokine IL‐8 from PMNs, and TNF‐α, IL‐6, IL‐12B, as well as the chemokines CCL2, CCL5 and CXCL1 from bone marrow‐derived macrophages previously treated with LPS (Recio et al., 2018; Suzuki et al., 2013). These were clearly GPR84‐mediated inflammatory responses as they were absent in cells isolated from homozygous GPR84 knockout mice and when such macrophages were treated with a selective GPR84 antagonist (see later) (Recio et al., 2018). In such LPS‐stimulated bone marrow‐derived macrophages, treatment with 6‐OAU enhanced phosphorylation of PKB (Akt) and the ERK1/2 and promoted p65 nuclear translocation (Recio et al., 2018). A series of analogues of 6‐OAU have also been generated by modifying the uracil head to incorporate alternative groups (Pillaiyar et al., 2018). Among 66 reported new derivatives in this study, a few have been considered in detail, based on improved potency, selectivity and metabolic stability. 6‐Hexylamino‐2,4(1*H*,3*H*)‐pyrimidinedione (PSB‐1584) (Figure 1) is reportedly some 100 times more potent than 6‐OAU in activating GPR84 (Pillaiyar et al., 2018). Homology modelling and docking studies have attempted to provide insights into the structural basis for the higher potency of uracil derivatives, particularly when compared with embelin and decanoic acid (Köse et al., 2020). These studies predicted a stronger interaction of uracil derivatives with residues Asn^357^ and Asn^104^, which may form part of the binding pocket of GPR84, as suggested initially by Köse et al. (2020) and Nikaido et al. (2015). However, while of interest, the dopamine D_3_ receptor bound by the antagonist eticlopride, which was used to develop the GPR84 homology model, is far removed from GPR84 in terms of overall sequence similarity, and thus, these predictions must be viewed as tentative. A tritium‐labelled form of PSB‐1584 ([^3^H]PSB‐1584) shows high affinity for GPR84 (*K*_D_ approx. 2 nM) (Table 1) (Köse et al., 2020), and [^3^H]PSB‐1584 has been used to quantify GPR84 in membrane preparations from cells and from human, rodent and bovine tissues (Köse et al., 2020). However, it may also bind to non‐GPR84 targets. Non‐transfected HEK293 cells do not express functional GPR84, but substantial levels of specific binding of [^3^H]PSB‐1584 were reported by Köse et al. (2020). Whilst it is well established that activation of GPR84 results in selective activation of *Pertussis* toxin‐sensitive, G_i_‐family G proteins, agonist‐occupied GPR84 can also interact with arrestins (Lucy et al., 2019; Pillaiyar et al., 2018; Southern et al., 2013; Zhang et al., 2016). Compounds that display different potency profiles at distinct endpoints (e.g., G‐protein activation and interaction with an arrestin) are described as displaying “bias” in relation to each other. Although this is normally compared with the action of the endogenous agonist(s), the fact that MCFAs are not fully accepted as the natural activators of GPR84 makes the selection of the appropriate reference agonist more complex in this case. However, the ligands 6‐((*p*‐chloro‐phenylethyl)amino)‐2,4(1*H*,3*H*)‐pyrimidinedione and 6‐((*p*‐bromo‐phenylethyl)amino)‐2,4(1*H*,3*H*)‐pyrimidinedione (PSB‐16434 and PSB‐17365, respectively; Figure 1) show a marked preference for activating G protein over interacting with arrestin (when compared with embelin) (Pillaiyar et al., 2018).

### 2.3 Alkylpyrimidine‐4,6‐diol derivatives

Based on a high‐throughput screening hit, a series of alkylpyrimidine‐4,6‐diol derivatives were identified as novel and potent GPR84 agonists (Liu et al., 2016; Zhang et al., 2016). These include 2‐(hexylthio)pyrimidine‐4,6‐diol (also referred to as ZQ‐16, Sundqvist et al., 2018; Zhang et al., 2016; 2‐HTP, Mancini et al., 2019; or compound 1, Al Mahmud et al., 2017; Liu et al., 2016) and 6‐nonylpyridine‐2,4‐diol, potentially the most potent agonist of GPR84 reported to date (Figure 1 and Table 1) (Liu et al., 2016; Zhang et al., 2016).

### 2.4 DL‐175 (3‐(2‐((4‐chloronaphthalen‐1‐yl)oxy)ethyl)pyridine 1‐oxide)

DL‐175 (3‐(2‐((4‐chloronaphthalen‐1‐yl)oxy)ethyl)pyridine 1‐oxide) was identified using a virtual screen based on comparisons with 6‐OAU (Figure 1). This would suggest it also should be an orthosteric agonist (Lucy et al., 2019). Using a recombinant cell system, DL‐175 showed comparable potency and efficacy to 6‐OAU in inhibiting cAMP accumulation; however, it was ineffective in an arrestin recruitment assay, indicating a marked bias for G‐protein signalling (Table 1) (Lucy et al., 2019). Interestingly, although not inducing signals in macrophages derived from GPR84 knockout mice (Lucy et al., 2019), DL‐175 generated a distinct signalling profile from 6‐OAU in both primary murine bone marrow‐derived macrophages and phorbol myristate acetate (PMA)‐differentiated human U937 macrophage‐like cells (Lucy et al., 2019). This may reflect the distinct bias of DL‐175 away from arrestin interactions as this would be anticipated to limit potential desensitisation. Interestingly, when cells were pretreated with CMPD101, a combined GPCR kinase GRK2 and GRK3 inhibitor (Ikeda, Kaneko, & Fujiwara, 2007), the response to 6‐OAU now resembled that of DL‐175 (Lucy et al., 2019). Finally, in contrast with 6‐OAU, DL‐175 failed to promote chemotaxis of M_1_‐polarised U937 macrophages (Lucy et al., 2019). Despite these interesting characteristics, DL‐175 is rapidly metabolised when exposed to mouse hepatocytes, suggesting that it will be of limited use for in vivo studies (Lucy et al., 2019).

## 3 ALLOSTERIC AGONISTS

### 3.1 DIM (3,3′‐methylenebis‐1*H*‐indole)

DIM (3,3′‐methylenebis‐1*H*‐indole) (Figure 1 and Table 1) is a metabolite produced in vivo from indole‐3‐carbinol, which is present at high levels in some vegetables including broccoli and kale (Wang, Schoene, Milner, & Kim, 2012). As with some of the orthosteric agonists described above, DIM can activate several targets including the aryl hydrocarbon (Yin, Chen, Mao, Wang, & Chen, 2012) and oestrogen (Marques et al., 2014) receptors. Although the mechanistic basis remains unclear, it also generates anti‐obesity effects in mice (Seo et al., 2011) and has anticancer activity both in vitro and in vivo (Fares, 2014; Firestone & Bjeldanes, 2003; Kiselev et al., 2014). It is unlikely to mediate these effects via activation of GPR84. Initially, DIM was described as a non‐lipid‐like GPR84 agonist by Takeda et al. (2003). However, the lack of structural relatedness to MCFAs and that it acts to enhance the actions of C10 as well as other orthosteric agonists at GPR84, defined DIM as behaving as a positive allosteric modulator (PAM) as well as a direct agonist (ago‐PAM) at GPR84 (Al Mahmud et al., 2017; Nikaido et al., 2015). As DIM is produced in vivo it has been suggested that it might enhance the potency of MCFAs sufficiently to allow circulating and local concentrations of MCFAs to activate GPR84 effectively. As noted earlier, the direct agonist effects of DIM are unaffected by mutagenesis of Arg^172^, hence confirming the binding site, or the route of entry to this binding site, to be distinct from the MCFAs and other orthosteric agonists (Al Mahmud et al., 2017). Although analysis of the positive allosteric interactions between DIM and MCFAs at GPR84 has allowed calculation that DIM binds (*K*_A_ range 5.6–6.9 μM) with some 200‐fold higher affinity than MCFAs (*K*_A_ range 0.17–0.53 mM) (Al Mahmud et al., 2017), it is still only a moderately potent activator. A more recent study confirmed DIM (and its derivatives) to be a positive allosteric modulator of GPR84 based on its ability to increase binding of [^3^H]PSB‐1584 (Köse et al., 2020). As expected from the concept of “probe dependence” of allosteric effects (Bartuzi, Kaczor, & Matosiuk, 2018), DIM displays different levels of co‐operativity with different orthosteric agonists of GPR84 (Al Mahmud et al., 2017) consistent with these binding and promoting receptor activation in somewhat different ways. As with 6‐OAU, Pillaiyar et al. (2017) and Pillaiyar et al. (2018) have performed extensive structure–activity studies designed to identify analogues with improved potency. This has been moderately successful. Both di(5‐fluoro‐1*H*‐indole‐3‐yl)methane (PSB‐15160) and di(5,7‐difluoro‐1*H*‐indole‐3‐yl)methane (PSB‐16671) (Figure 1) are somewhat more potent than DIM but display bias, compared with DIM, towards G_i_‐mediated AC inhibition over arrestin recruitment (Pillaiyar et al., 2017). While both PSB‐15160 and PSB‐16671 are markedly selective for GPR84, compared with the long‐chain free fatty acid receptors FFA1 and FFA4 (Pillaiyar et al., 2017), at least PSB‐16671 possesses currently undefined off‐target liabilities. For example, PSB‐16671 was equally effective in activating G proteins in mouse bone marrow‐derived neutrophils isolated from both wild‐type and GPR84 knockout mice (Mancini et al., 2019). As the parental ligand DIM did not display this characteristic, then the markedly greater efficacy of PSB‐16671 in membranes from a mouse GPR84‐expressing HEK293‐derived cell line when measuring binding of [^35^S]GTPγS is most likely a reflection of this off‐target effect rather than substantially higher efficacy of PSB‐16671 over DIM at GPR84 (although a somewhat higher level of efficacy of PSB‐16671, compared with DIM, was observed in a recombinant cell system [Flp‐In T‐REx‐293 cells] expressing human GPR84; Mancini et al., 2019). This conclusion is supported by the observation that a GPR84 antagonist (see later) only partly blocked the effect of PSB‐16671 in such membranes, whereas the stimulation produced by DIM was fully attenuated by the same antagonist (Mancini et al., 2019). Moreover, the same GPR84 antagonist fully blocked [^35^S]GTPγS binding in membranes prepared from human neutrophils indicating on‐target effects (Mancini et al., 2019). An obvious feature across the range of GPR84 agonists, whether potentially orthosteric or allosteric, is that when effects specifically reflecting activation of GPR84 are compared, the potencies of compounds at human and mouse receptors are very close. This may not be considered particularly surprising given the noted high sequence similarity in the transmembrane and extracellular regions of these orthologues, but interestingly, this is not true for the best characterised class of currently described GPR84 antagonists (Mancini et al., 2019).

## 4 ANTAGONISTS

The best characterised GPR84 antagonists are from a series of dihydropyrimidinoisoquinolinones described initially by Labéguère, Alvey, Newsome, Saniere, and Fletcher (2014) (Table 2). A member of this series, 9‐cyclopropylethynyl‐2‐((*S*)‐1‐[1,4]dioxan‐2‐ylmethoxy)‐6,7‐dihydropyrimido[6,1‐*a*]isoquinolin‐4‐one (GLPG1205) (Figure 1 and Table 2), progressed into first‐in‐human clinical trials for the potential treatment of ulcerative colitis but failed to achieve defined levels of efficacy despite reducing disease activity and neutrophil infiltration in a mouse model of chronic inflammatory bowel disease (IBD), induced by dextran sodium sulphate (Labéguère et al., 2020). These ligands have the characteristics of non‐competitive antagonists of GPR84 (as least as defined by the nature of their blockade of the action of “orthosteric” agonists as defined above) (Al Mahmud et al., 2017; Labéguère et al., 2020) and also act as non‐competitive blockers of the effects of the group of allosteric agonists represented by DIM (Al Mahmud et al., 2017). In addition to a number of compounds from this series that have been shown to block effects of GPR84 agonists in a variety of settings (Table 2) (Al Mahmud et al., 2017; Mancini et al., 2019; Sundqvist et al., 2018), [^3^H]G9543 (a tritiated version of “compound 38” described in Labéguère et al., 2020) has been instrumental in demonstrating that pro‐inflammatory signals such as exposure to LPS act to up‐regulate levels of the receptor at protein as well as mRNA level (Mancini et al., 2019). In agreement with the characterisation of these dihydropyrimidinoisoquinolinones as non‐competitive antagonists, although different compounds from the same series are able to fully displace the specific binding of [^3^H]G9543 from GPR84, neither orthosteric nor allosteric agonists can do so (Al Mahmud et al., 2017; Labéguère et al., 2020). Unlike the agonists, this class of antagonist shows somewhat lower (between 20‐fold and 70‐fold in various settings and reports) affinity at mouse GPR84, compared with the human orthologue (Labéguère et al., 2020; Mancini et al., 2019). In practice, this has meant that [^3^H]G9543 is not useful for quantification of levels of expression of GPR84 in mouse tissues or mouse‐derived cell lines. The basis for this difference remains undefined but once again, as with the orientation of MCFAs, is likely be to fully understood only when appropriate atomic‐level structures become available. Currently, the only described compound with GPR84 antagonist activity that is likely to act in an orthosteric fashion is PBI‐4050 (2‐(3‐pentylphenyl)acetic acid) (Parker et al., 2017) (Figure 1 and Table 2) as this is a fatty acid derivative. Although it is able to block GPR84, its affinity at the receptor is very modest, and hence, formal proof of a competitive orthosteric mode of action is difficult to achieve. Moreover, although PBI‐4050 is currently the subject of a variety of clinical trials (see Khalil et al., 2019), it clearly has actions at receptors other than GPR84. Indeed, Gagnon et al. (2018) noted its ability to agonise the long‐chain fatty acid receptor FFA1, and our own studies have noted effects also at the short‐chain fatty acid receptor FFA2.

**TABLE 2:** Antagonist ligands with affinity at GPR84

Compound name	Potency (IC_50_)	Model system (in vitro, ex vivo)	Comment	References
**Orthosteric**				
PBI‐4050	**Human:** 0.4 mM (vs. sodium decanoate)^b^ 0.21 mM (vs. embelin)^b^	‐Recombinant system ‐Human dermal fibroblasts ‐Human epithelial proximal tubule cells ‐Peritoneal mouse macrophages ‐Human podocytes ‐Human hepatic stellate cells ‐Human lung fibroblasts		Parker et al., 2017 Gagnon et al., 2018 Grouix et al., 2018 Li et al., 2018 Khalil et al., 2019 Nguyen et al., 2020
**Allosteric**				
Compound 107	**Human:** 7.24–13.8 nM (vs. 2‐HTP)^a^ 0.89–4.8 nM (vs. PSB‐16671)^a^ **Mouse:** 125.9–316.2 nM (vs. 2‐HTP)^a^ 67.61–144.5 nM (vs. PSB16671)^a^	‐Recombinant system ‐THP‐1 monocytes ‐RAW264.7 cells ‐Mouse bone marrow‐derived neutrophils ‐Human blood‐derived neutrophils	A tritium‐labelled form of an antagonist compound from the same series of 107 is available ([^3^H]G9543)^c^	Labéguère et al., 2014 Al Mahmud et al., 2017 Mancini et al., 2019 Labéguère et al., 2020
GLPG1205	**Human:** 54 nM (vs. DIM)^a^	‐Recombinant system ‐Human blood‐derived neutrophils ‐Rat blood‐derived neutrophils ‐Dog blood‐derived neutrophils ‐Human monocyte‐derived macrophages	From the same series as 107: in clinical trials in patients with idiopathic pulmonary fibrosis	Labéguère et al., 2014 Labéguère et al., 2020 Vermeire et al., 2017 Sundqvist et al., 2018

^a^ Potency values were generated using [^35^S]GTPγS assay.

^b^ Potency values were generated using BRET activation biosensor assay.

^c^ Al Mahmud et al. (2017), Mancini et al. (2019), and Labéguère et al. (2020).

## 5 EXPRESSION PROFILE OF GPR84

**FIGURE 2:**
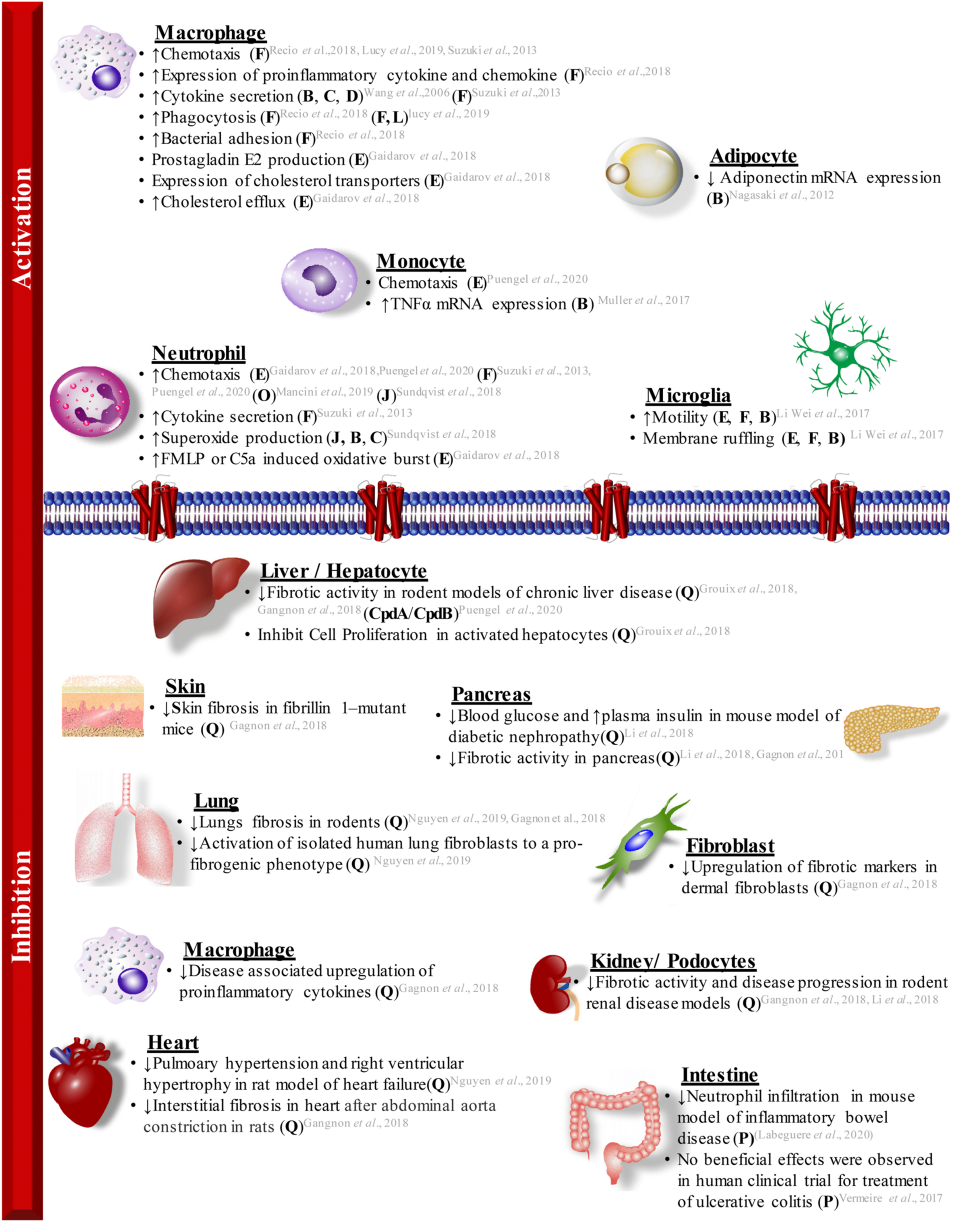
GPR84 modulation in inflammatory conditions. Upper panel: Activation of GPR84 by orthosteric and allosteric ligands generates pro‐inflammatory responses in different cell types. Lower panel: GPR84 inhibition reduces responses associated with inflammation, fibrosis, and metabolic diseases in multiple cell types and tissues (letters indicate use of ligands illustrated in Figure 1)

Transcriptome studies have revealed that GPR84 is expressed primarily in a subset of peripheral immune cells (Figure 2). Following initial identification on peripheral blood leukocytes (Yousefi et al., 2001), subsequent studies confirmed high expression of GPR84 mRNA in both human and mouse neutrophils and macrophages (Bouchard, Page, Bedard, Tremblay, & Vallieres, 2007; Lattin et al., 2008; Sundqvist et al., 2018; Suzuki et al., 2013; Wang et al., 2006). Relatively lower levels of GPR84 mRNA transcript have been reported in other immune cells, including dendritic cells, T cells, and B cells (Suzuki et al., 2013; Wang et al., 2006), and in microglial cells in the CNS (Gautier et al., 2012; Hickman et al., 2013; Recio et al., 2018). Although limited, expression has been detected outside the immune system. For example, some studies have found GPR84 mRNA in adipocytes, epithelial cells, fibroblast, and podocytes (Abdel‐Aziz et al., 2016; Gagnon et al., 2018; Nagasaki et al., 2012). Receptor expression in many of these tissues could possibly be due to immune cells resident in these tissues, but this has yet not been confirmed.

## 6 GPR84 UP‐REGULATION IS ASSOCIATED WITH PRO‐INFLAMMATORY CONDITIONS

Up‐regulation of GPR84 in immune cells at both mRNA and protein levels has been reported in response to inflammatory conditions and stimuli. Recent examples of such observations include that treatment of human and mouse macrophages and the human monocytic cell line THP‐1 with the TLR4 activator LPS caused up‐regulation of GPR84 expression (Mancini et al., 2019; Recio et al., 2018). Similar outcomes are obtained in vivo, where up‐regulation of GPR84 mRNA expression was observed during acute inflammation in a mouse model of endotoxemia. Additionally, up‐regulation was also observed in models of chronic inflammation, including diabetes and atherosclerosis (Recio et al., 2018). Consistent with the notion that GPR84 is a pro‐inflammatory receptor, increase in GPR84 expression has also been reported in colonic tissues and blood samples of patients with IBD (Arijs et al., 2011; Planell et al., 2017). As previously noted, it remains unclear to what extent GPR84 up‐regulation in these tissues can be attributed to immune cell infiltration. However, the studies of Arijs et al. (2011) have been quoted as a driver leading to the assessment of whether blockade of GPR84 with the antagonist GLPG1205 might improve clinical observations in patients with ulcerative colitis (Labéguère et al., 2020).

Although up‐regulation of GPR84 has been primarily studied in immune cells, a limited number of studies have also observed up‐regulation in fat cells (Figure 2). GPR84 expression in human adipocytes is significantly up‐regulated following acute exposure to IL‐33, TNF‐α, or IL‐1β. This increase, however, was reported to be transitory, since the increase in receptor mRNA expression was not present following 24 h of exposure (Muredda, Kepczynska, Zaibi, Alomar, & Trayhurn, 2018; Zaibi, Kepczynska, Harikumar, Alomar, & Trayhurn, 2018). Similar observations have been made with treatment of mouse 3T3‐L1 adipocytes with TNF‐α and LPS and in human adipose‐derived stem cells (Nagasaki et al., 2012). Up‐regulation of GPR84 mRNA was also observed in vivo, specifically in fat pads of mice fed with a high‐fat diet. Interestingly, a correlation was reported between receptor mRNA up‐regulation and increase in specific staining for macrophages, suggesting infiltration of immune cells as the likely cause of receptor up‐regulation (Nagasaki et al., 2012). An increase in GPR84 mRNA transcripts has also been reported in liver biopsies of patients with non‐alcoholic fatty liver disease (NAFLD). This increase, however, is associated with inflammation rather than fat accumulation in the liver (Puengel et al., 2020). In line with other reports, higher levels of GPR84 mRNA have been observed in fibroblasts, podocytes, proximal tubule epithelial cells, and macrophages under fibrotic conditions (Gagnon et al., 2018; Grouix et al., 2018; Li et al., 2018). Interestingly, this up‐regulation is also accompanied by up‐regulation of various pro‐fibrotic and inflammatory biomarkers, and treatment with PBI‐4050 significantly reduced the expression of these markers (Gagnon et al., 2018; Grouix et al., 2018; Li et al., 2018). Such observations underpin the clinical trials of both PBI‐4050 (Khalil et al., 2019) and more recently GLPG1205 (Labéguère et al., 2020) in idiopathic pulmonary fibrosis. Unsurprisingly, promising initial results have promoted ideas that blockade of GPR84 might be of more general use in a wider range of fibrotic conditions (Nguyen et al., 2020; Wojciechowicz & Ma'ayan, 2020). The exact mechanism responsible for disease‐induced GPR84 up‐regulation in many in vivo studies remains to be identified. Nevertheless, these studies further highlight the potential role of the receptor in disease conditions primarily associated with inflammation. Furthermore, observations of GPR84 up‐regulation are not limited to peripheral diseases but have also been demonstrated in neuroinflammatory conditions in the CNS. Although low‐level expression of GPR84 was reported in the brain of healthy adult mice in early studies (Bouchard et al., 2007), inflammatory stimuli induce significant up‐regulation in CNS microglia, for example, in a model of endotoxic shock (Audoy‐Remus et al., 2015). TNF‐α and IL‐1 have been suggested to play a key role in GPR84 up‐regulation, as mice lacking these molecules show reduced expression of GPR84 in the cerebral cortex (Bouchard et al., 2007). Higher levels of receptor mRNA expression are also reported in other animal disease models affecting the CNS, these include experimental autoimmune encephalomyelitis, a model of multiple sclerosis (Bouchard et al., 2007), cuprizone‐induced demyelination and axotomy (Bedard, Tremblay, Chernomoretz, & Vallieres, 2007), and in a murine model of Alzheimer's disease (APP‐PS1) (Audoy‐Remus et al., 2015). Many of these reports now are now ready to be followed up, with the availability of a substantially wider range of pharmacological tools, than at the time of the original observations.

Not surprisingly, given the strong up‐regulation of GPR84 mRNA in such conditions, it has been suggested that GPR84 could also be a useful marker for activation of glial cells following CNS damage. In addition to up‐regulation of GPR84 mRNA following TLR stimulation of microglia and astrocytes in vitro, GPR84 up‐regulation has also been noted in vivo, for example, following La Crosse virus infection, which results in considerable neuronal cell death and inflammation in the CNS (Madeddu et al., 2015). Similarly, Gamo et al. (2008) also reported up‐regulation of GPR84 mRNA in mouse brain following hypoglossal axotomy (motor nerve injury). Higher expression of GPR84 observed during inflammation and injury also supports the premise that targeting GPR84 might have therapeutic value in neuroinflammatory CNS disorders.

## 7 MODULATING GPR84 ACTIVITY: FURTHER THERAPEUTIC OPPORTUNITIES?

As highlighted above, improved availability of various GPR84 ligands as well as GPR84 knockout mice has enhanced understanding of receptor function and is now allowing assessment of whether blockade of GPR84 might have therapeutic utility in various inflammatory and fibrotic conditions. However, a series of studies have also made potential links with other conditions including pain, atherosclerosis, and even metabolic disorders. A key study directly exploring the role of GPR84 in nociceptive transmission reported lack of pain hypersensitivity in GPR84 knockout mice following peripheral nerve injury. Consistent with this observation, up‐regulation of GPR84 mRNA and anti‐inflammatory macrophage markers (*Arg‐1* and cytokine *Il‐10*) were also reported in spinal cord and sciatic nerve after partial sciatic nerve ligation (PNL) (Nicol et al., 2015). This study provides a rationale for further investigating the potential role of GPR84 in chronic pain states using low MW antagonists in mouse models to relieve inflammatory pain. Despite this, in vivo studies have demonstrated that antagonism of GPR84 might be harmful in aspects of neurodegeneration. In APP‐PS1, lack of GPR84 expression had significant effect on disease progression, as APP‐PS1 GPR84‐deficient mice were shown to have reduced β‐amyloid‐induced microgliosis. These mice also demonstrated higher impairment of dendritic integrity and cognitive function (Audoy‐Remus et al., 2015). Although these data highlight the potentially deleterious effects of GPR84 antagonism, it is not clear whether activating GPR84 may be beneficial in slowing disease progression and therefore further study is needed to clarify the role(s) of this GPCR.

A limited number of studies have also shown how activation of GPR84 may be beneficial in metabolic diseases. Such studies have highlighted a possible role of GPR84 in lipid metabolism. Higher levels of hepatic triglyceride were reported in GPR84 knockout mice maintained on a MCFA‐enriched diet. This study, however, did not find any influence of GPR84 activity on body weight or glucose tolerance (Du Toit et al., 2018). Conversely, another group reported a role of GPR84, in both glucose tolerance and reduced insulin plasma levels in mice and pancreatic islets (Montgomery et al., 2019). Interestingly, this study also emphasised how GPR84 might be involved in mitochondrial metabolism. Loss of GPR84 led to impaired mitochondria and increased oxidative stress. Despite the observed differences in measures of glucose tolerance, these findings support the notion that activation of GPR84 may be beneficial as a therapeutic strategy for metabolic dysfunction associated with obesity. This is consistent with another study reporting anti‐atherosclerotic effects of GPR84 agonism. Activation of GPR84 by embelin leads to release of PGE_2_ from human macrophages. GPR84 agonism also up‐regulates expression of the cholesterol transporters ABCA1 and ABCG1 and induces a significant increase in apolipoprotein A‐I‐mediated efflux of cholesterol (Gaidarov et al., 2018). As opposed to inflammatory conditions, where inhibiting GPR84 function may serve a potential therapeutic benefit, agonism of GPR84 may be beneficial for its anti‐atherosclerotic properties.

## 8 CONCLUSIONS AND FUTURE PERSPECTIVES

Although identified and subsequently shown to respond to MCFAs more than 15 years ago, recent years have provided substantial advances in understanding of the basic biology and regulation of GPR84, and these have resulted in ongoing clinical trials targeting this receptor in fibrotic conditions. However, there are many areas where additional information would greatly facilitate the drive towards clinical validation. These include direct structural information of the mode(s) of binding of various classes of ligands to the receptor and indeed the availability of a broader range of, particularly antagonist, ligands. Although clear definition of the true endogenous regulator(s) of this receptor would be welcome, this is unlikely to restrict efforts to target this receptor, but recent indications of distinct functional outcomes of agonist ligands with substantial signalling “bias” are likely to promote efforts to understand, in greater detail, ways to harness the differences between actions of distinct agonists also in a therapeutic context. Equally, although measures of regulated mRNA levels have been insightful, the ability to quantify such effects more directly at the protein level, whether by the development of improved radiopharmaceuticals, including potential PET ligands, or indeed via the availability of suitable antibody tools, would offer many novel opportunities. There is certainly potential for inflammation‐induced up‐regulation of GPR84 mRNA to provide a sensitive and valuable preclinical biomarker, and as well as peripheral disorders, blockade of GPR84 may be of value in the treatment of neuroinflammatory conditions in the CNS. Further insights into this receptor are now likely to emerge rapidly.

### 8.1 Nomenclature of targets and ligands

Key protein targets and ligands in this article are hyperlinked to corresponding entries in the IUPHAR/BPS Guide to PHARMACOLOGY (http://www.guidetopharmacology.org) and are permanently archived in the Concise Guide to PHARMACOLOGY 2019/20 (Alexander, Christopoulos et al., 2019; Alexander, Cidlowski et al., 2019; Alexander, Fabbro et al., 2019a, 2019b; Alexander, Kelly et al., 2019a, 2019b).

## CONFLICT OF INTEREST

The authors declare no conflicts of interest.
